# Restricted gene flow at the micro- and macro-geographical scale in marble trout based on mtDNA and microsatellite polymorphism

**DOI:** 10.1186/1742-9994-8-7

**Published:** 2011-04-14

**Authors:** José M Pujolar, Alvise N Lucarda, Mauro Simonato, Tomaso Patarnello

**Affiliations:** 1 Dipartimento di Biologia, Università di Padova, 35121 Padova PD, Italy; 2 Dipartimento di Produzioni Animali, Epidemiologia ed Ecologia, Università di Turin, 10095 Grugliasco TO, Italy; 3 Dipartimento di Agronomia Ambientale e Produzioni Vegetali, Agripolis, Università di Padova, 35020 Legnaro PD, Italy; 4 Dipartimento di Sanità Pubblica, Patologia Comparata ed Igiene Veterinaria, Agripolis, Università di Padova, 35020 Legnaro PD, Italy

## Abstract

**Background:**

The genetic structure of the marble trout *Salmo trutta marmoratus*, an endemic salmonid of northern Italy and the Balkan peninsula, was explored at the macro- and micro-scale level using a combination of mitochondrial DNA (mtDNA) and microsatellite data.

**Results:**

Sequence variation in the mitochondrial control region showed the presence of nonindigenous haplotypes indicative of introgression from brown trout into marble trout. This was confirmed using microsatellite markers, which showed a higher introgression at nuclear level. Microsatellite loci revealed a strong genetic differentiation across the geographical range of marble trout, which suggests restricted gene flow both at the micro-geographic (within rivers) and macro-geographic (among river systems) scale. A pattern of Isolation-by-Distance was found, in which genetic samples were correlated with hydrographic distances. A general West-to-East partition of the microsatellite polymorphism was observed, which was supported by the geographic distribution of mitochondrial haplotypes.

**Conclusion:**

While introgression at both mitochondrial and nuclear level is unlikely to result from natural migration and might be the consequence of current restocking practices, the pattern of genetic substructuring found at microsatellites has been likely shaped by historical colonization patterns determined by the geological evolution of the hydrographic networks.

## Introduction

Freshwater fish species show a greater average degree of genetic differentiation among locations than marine species, often resulting from the isolation of fish populations among drainages [1]. The marked zoogeography produced by the historical patterns of isolation among drainages is subjected to the continuous remodeling of the river drainage and to climatic fluctuations of which the glacial/interglacial periods of the Pleistocene played a crucial role [2]. Species respond actively to fluctuations in their natural range, with hydrographic networks being used for colonization and for retreat throughout geological times. In the case of salmonids, anadromy should increase gene flow among river basins [3]. However, Bernatchez et al. [4] reported significant heterogeneity at the mitochondrial DNA level among populations of the pan-European brown trout *Salmo trutta*, which lead to the definition of five distinct evolutionary lineages: four of them, the Adriatic (AD), the Atlantic (AT), the Danube (DA) and the Mediterranean (ME) lineage correspond to the major drainage basins, whereas the fifth lineage is represented by the marble trout (MA). Examination of the evolutionary relationships among the five trout lineages suggested a scenario in which the most ancient phylogeographic separation resulted from allopatric isolation in the Pleistocene between the three major European drainages, Atlantic (AT lineage), Danube (DA lineage) and Mediterranean (AD, MA and ME lineages), followed by later divergence within the Mediterranean basin linked to the existence of glacial refugia that originated the AD, MA and ME lineages [5]. More recently, a sixth lineage has been described, restricted to the Duero river in the Iberian Peninsula [6]. Complex genetic structures exist in many trout species, resulting from multiple colonization episodes by distinct lineages and secondary intergradiations, with restricted gene flow and extreme genetic drift producing deep genetic divergences [7-11].

The marble trout is characterized by a particular marbled colour pattern and different biological and ecological traits such as a very large body size (up to 1 m in length and 25 kg in weight) and the complete lack of black spots on the lateral side of the body. It has a restricted native geographic distribution within the Adriatic basin, found on the alpine part of the river Po in Northern Italy, former Yugoslavia and Albania [12]. The taxonomic status of marble trout is controversial, being recognized either as a separate species *Salmo marmoratus *[13-16] or as a sub-species *Salmo trutta marmoratus *within the *Salmo trutta *complex [17,18].

The existence of marble trout has been seriously threatened by the introduction of nonindigenous brown trout throughout its natural range by means of massive restocking practices [19]. Hybridization between marble and brown trout has been so extensive that hybrids now dominate most rivers, and typical marble trout forms are rare and limited to secluded headwater streams. Molecular data confirm a high level of introgression in the western part of the Po river in Northern Italy [17,18], the Soca river system in Slovenia [14-16], and recently, the Adige river system in South Tyrol [20,21], although all studies also reported pure populations of marble trout in headwaters of all river systems.

Up to now, molecular genetic studies on marble trout have focused on species identification using mitochondrial DNA haplotypes and assessing the rate of introgression among lineages in the *Salmo trutta *complex, while examination of the population genetic structure of marble trout has been mostly neglected. Using allozymes, Giuffra et al. [18] found no apparent differences among marble trout samples collected along tributaries of the Po river system in Northern Italy, which contrasted with the fixed or nearly fixed differences for alternate alleles at 8 loci found between marble and brown trout (Nei's genetic distance of 0.16-0.18). However, extreme genetic heterogeneity has been found among remnant marble trout populations from the Soca river system in Slovenia using microsatellites, with a global *F*_ST _= 0.66 (p < 0.001) and all pairwise *F*_ST _ranging from 0.31 to 0.88 [16].

**Figure 1:**
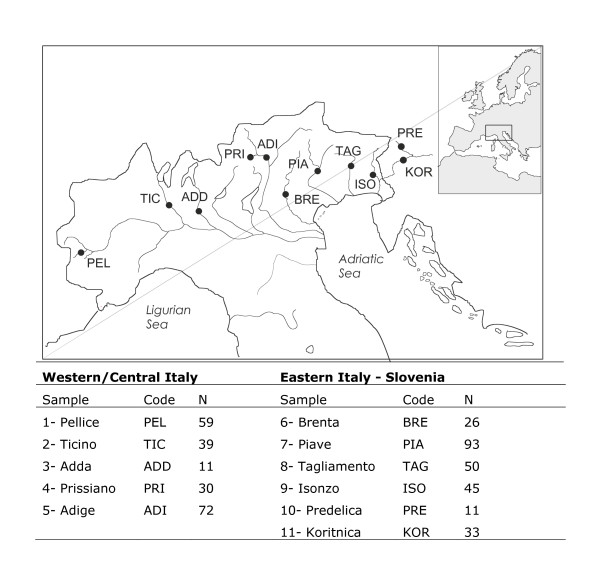
**Sampling locations of marble trout**.

The aim of this study is to perform the most extensive genetic survey to date of marble trout populations sampled throughout its geographic distribution, including tributaries of the Po and Soca (known as Isonzo in Italy) rivers, the two drainage systems in which the species is most prominently found (Figure 1). A total of 429 specimens from 11 rivers were screened for genetic variation at mitochondrial and microsatellite markers in order to disentangle the native patterns of population genetic structure and genetic diversity of marble trout at the micro-geographic (within rivers) and macro-geographic (among river systems) scale.

## Results

A total of seven mtDNA haplotypes were found in our dataset (Table 1), three of which were identified as marble trout haplotypes, while the remaining four haplotypes were identified as typical of brown trout [4]. Two out of the three marble trout haplotypes (MA-s1 and MA-s2) were described in previous studies [4,5,17], while haplotype MA-s4 is reported here for the first time. Using haplotype MA-s1 from Bernatchez [5] as reference, haplotype MA-s4 differs by a single nucleotide (G instead of A at position 278). Non-marble trout haplotypes were observed in most locations, which suggests introgression of brown trout into marble trout. Nevertheless, brown trout haplotypes were completely absent in Pellice and Predelica, and were present in low frequencies (<4%) in Prissiano, Adige, Piave and Tagliamento. The Atlantic haplotype AT-s1 (considered the allochthonous one) was found in high frequencies in the samples Koritnica (42%), Isonzo (11%), Adda (9%), Ticino (8%) and Brenta (8%). The haplotype DA-s1, usually associated with brown trout from the Danube drainage system, was very frequent in Ticino (23%) and Adda (45%).

**Table 1:** Frequencies of marble and brown trout mitochondrial haplotypes across samples.

	Mitochondrial DNA							Microsatellite		
	**Marble trout**			**Brown trout**				**Marble trout**	**>10% Brown trout**	**Introgression rate (%)**

	**MA-S1**	**MA-S2**	**MA-S4**	**AT-S1**	**DA-S1**	**DA-S2**	**AD-S1**			

**PEL**		59						55	4	3.3
**TIC**	6	20	1	3	9			15	24	18.5
**ADD**		4	1	1	5			2	9	33.0
**PRI**		29		1				22	8	8.6
**ADI**		71					1	69	3	3.7
**BRE**		24		2				14	12	14.9
**PIA**	6	86		1				79	14	7.5
**TAG**	4	44				2		26	24	12.4
**ISO**	25	15		5				14	31	12.9
**PRE**	11							11	0	0.5
**KOR**	18			14	1			0	33	60.9

**Chisone**				23				0	23	0.5

Introgression at microsatellite loci is presented as number of individuals with >10% of brown trout material on the basis of STRUCTURE analysis. Introgression rate (average value of the admixture coefficient) is provided for each location. A pure brown trout sample (Chisone) is included for comparison. Samples labelled as in Figure 1.

**Figure 2:**
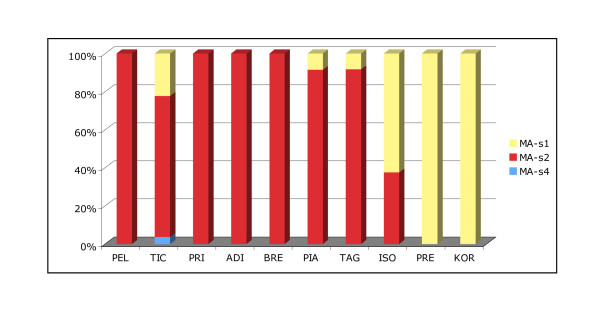
**Frequency of marble trout haplotypes (MA-s1, MA-s2 and MA-s4) across samples, labelled as in Figure 1**.

Comparison of mtDNA haplotype frequencies showed highly significant differences across sampling locations (p < 0.001). The genetic differentiation is essentially due to the differential distribution of haplotypes Ma-s1 and MA-s2: haplotype Ma-s2 appears to be fixed in the most western locations (Pellice, Prissiano, Adige, Brenta), decreases progressively in frequencies moving toward eastern locations (Piave, Tagliamento, Isonzo), and is replaced by haplotype MA-s1 in the two Slovenian locations (Predelica, Koritnica). The only exception was Ticino, the second most western sample, with 22% haplotype MA-s1 individuals (Figure 2).

**Figure 3:**
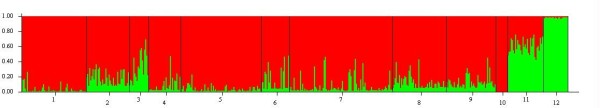
**Admixture analysis using STRUCTURE, in which individuals were assigned on the basis of the most likely K (in this case, K = 2)**. Populations are numbered from 1 to 11 following the order in Figure 1. Population 12 is a pure brown trout population (Chisone). Each vertical bar represents one individual, partitioned into two segments according to the proportion of marble trout (red) and brown trout (green).

A higher level of introgression was observed at the microsatellite level. When analyzing all marble trout samples together with one brown trout sample (Chisone) using STRUCTURE, a scenario with two clusters (K = 2) corresponding to the two taxa was the most likely. When using a 10% expected brown trout introgression as threshold, introgressed individuals were found at all locations except Predelica in Slovenia (Table 1; Figure 3). Introgression rate was low (3-9%) in Pellice, Prissiano, Adige and Piave, which agrees with a low introgression at the mitochondrial level in those locations. Correspondingly, introgression at the microsatellite level was notably high in Adda (33%) and Koritnica (60.9%), which were also the locations with the highest introgression at the mitochondrial level.

Values of genetic variability at microsatellite loci are summarized in Table 2. Genetic variability was clearly correlated with introgression level, with the highest genetic diversities observed in the locations with the highest introgression (Adda, Koritnica). Lower diversities were observed in the locations with low introgression, notably Predelica (the only 100% pure marble trout population identified at microsatellites) where only a total of 7 alleles were observed at 5 loci (*AR *= 1.4).

**Table 2:** Summary of genetic variability at microsatellite loci across marble trout samples including number of individuals (N), observed (*H*_*o*_) and expected (*H*_*e*_) heterozygosities and allelic richness (*AR*) considering a) all individuals and b) only non-introgressed individuals.

Sample	N	***H***_***o***_	***H***_***e***_	*AR*
**a)**				
**PEL**	59	0.177 (0.175)	0.193 (0.183)	2.548 (1.193)
**TIC**	39	0.550 (0.228)	0.549 (0.257)	4.968 (2.549)
**ADD**	11	0.600 (0.133)	0.612 (0.180)	5.105 (2.273)
**PRI**	30	0.366 (0.217)	0.389 (0.233)	3.428 (1.253)
**ADI**	72	0.394 (0.310)	0.399 (0.313)	3.580 (1.972)
**BRE**	26	0.475 (0.309)	0.458 (0.284)	4.134 (1.741)
**PIA**	93	0.298 (0.265)	0.365 (0.286)	3.715 (2.092)
**TAG**	50	0.331 (0.229)	0.401 (0.285)	3.623 (1.783)
**ISO**	45	0.370 (0.253)	0.426 (0.274)	3.967 (1.902)
**PRE**	11	0.055 (0.122)	0.080 (0.113)	1.400 (0.826)
**KOR**	33	0.624 (0.130)	0.707 (0.121)	5.366 (2.273)
**Chisone**	23	0.625 (0.250)	0.749 (0.047)	7.513 (2.309)

**b)**				
**PEL**	55	0.153 (0.159)	0.164 (0.180)	2.262 (1.006)
**TIC**	15	0.360 (0.332)	0.333 (0.321)	2.914 (1.155)
**PRI**	22	0.276 (0.250)	0.290 (0.261)	2.592 (1.099)
**ADI**	69	0.390 (0.317)	0.388 (0.316)	3.389 (1.772)
**BRE**	14	0.346 (0.483)	0.283 (0.387)	2.392 (1.541)
**PIA**	79	0.248 (0.300)	0.290 (0.319)	3.051 (1.233)
**TAG**	26	0.249 (0.337)	0.275 (0.345)	2.474 (1.201)
**ISO**	14	0.200 (0.369)	0.223 (0.320)	2.306 (1.366)
**PRE**	11	0.055 (0.122)	0.080 (0.113)	1.400 (0.826)

A pure brown trout sample (Chisone) is included for comparison. Standard deviation in parentheses. Samples labelled as in Figure 1.

In order to estimate the native patterns of population genetic structure and gene diversity, data was re-analyzed removing all individuals with >10% expected introgression. As a consequence, Adda (reduced to only two individuals) and Koritnica (all individuals introgressed) were excluded from the analysis. The effect of removing introgressed individuals was a expected drop in genetic diversity (Table 2). Heterozygosities were similar across samples except for Predelica, which showed a 2-4-fold lower heterozygosity (H_e _= 0.080) in comparison with the rest of samples (*H*_e _= 0.164-0.388). Following Bonferroni correction, only one out of 45 tests departed significantly from HWE (locus Str85 at Piave). The software MICROCHECKER showed no evidence for scoring errors due to stuttering or large alle dropout. An homozygote excess was found at locus Str85, which might be attributable to the presence of null alleles, at Adige, Isonzo and Piave, but not at the rest of samples. No linkage disequilibrium was observed between any pair of loci after Bonferroni correction.

Since our study was carried out with a limited number of microsatellite markers, statistical power to detect genetic divergence was assessed with POWSIM prior to the genetic differentiation analysis. Simulations using our empirical microsatellite data, taking into account the sample size for each population, and a wide range of predefined *F*_ST _values (0.001, 0.005, 0.01, 0.05, 0.1) showed that our markers have enough statistical power to detect *F*_ST _values > 0.005 (p = 1.000). This suggests that our markers can provide a reasonably accurate picture of the genetic population structure of marble trout.

**Figure 4:**
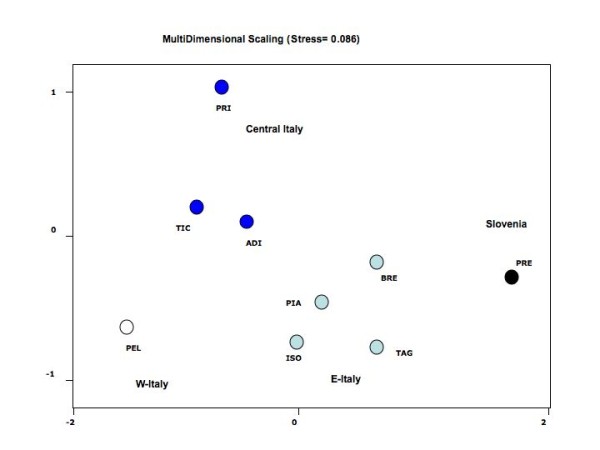
**Plots from Multi-Dimensional Scaling analysis based on Cavalli-Sforza and Edwards (1969) chord distance**. Samples labelled as in Figure 1.

**Figure 5:**
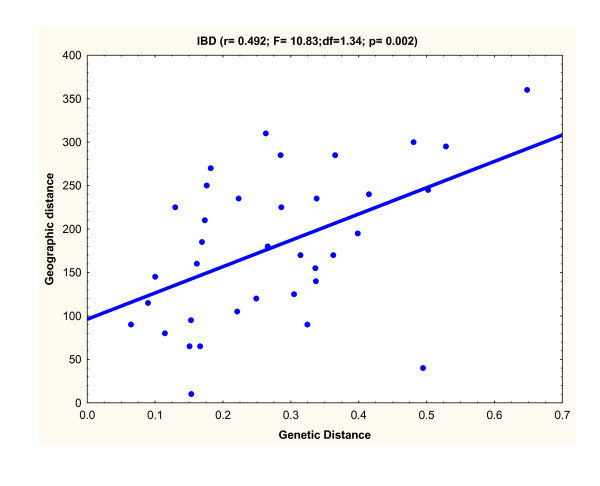
**Correlation between genetic distance (*F***_**ST **_**/1- *F***_**ST**_**) and hydrographic distance**.

When investigating the genetic structure of native populations (introgressed individuals excluded), all pairwise *F*_ST _comparisons were highly significant (p < 0.001; Table 3), with an average pairwise *F*_ST _value of 0.276. The highest pairwise value was found between the two most distant locations, Pellice and Predelica (*F*_ST _= 0.648). Similarly, pairwise genetic distances between population pairs were all highly significant (p < 0.001; Table 3). When representing genetic distances using Multi-dimensional Scaling analysis (Figure 4), a sequential distribution of samples was obtained across the horizontal axis of the plot that appears in agreement with the West-to-East geographic distribution of the sampling locations (Figure 1). A Mantel test showed a significant correlation between linearized *F*_ST _and waterway distances (r = 0.492; F = 10.83; df = 1.34; p = 0.002), which suggests an Isolation-by-Distance pattern (Figure 5). AMOVA analysis under a hydrogeographic hierarchical model partitioned significantly within river basins but not among rivers (*F*_ST _= 0.238; p = 0.000; *F*_SC _= 0.236; p = 0.000 *F*_CT _= 0.003; p = 0.385), which might be due to the deep genetic break between Pellice and Ticino in the Po river and Isonzo and Predelica in the Soca river. In concordance with the MDS analysis, a hierarchical AMOVA partitioned significantly among groups only when considering four groupings corresponding to Western Italy (Pellice), Central Italy (Ticino, Prissiano, Adige), Eastern Italy (Piave, Brenta, Isonzo, Tagliamento) and Slovenia (Predelica) (Table 4). A Structure analysis run with an admixed model of correlated allele frequencies suggested a scenario with four clusters (K = 4) corresponding practically to the AMOVA groups as the most likely (data not shown). Re-examination of population genetic structure including introgressed individuals similarly showed a highly significant overall genetic differentiation (*F*_ST _= 0.226), a significant Isolation-by-Distance pattern (r = 0.399; F = 7.26; df = 1.43; p = 0.011), and the same sequential distribution of samples following the West-to-East distribution of the sampling locations.

**Table 3:** Pairwise *F*_ST _estimates (above diagonal) and genetic distances (D_CE_, below diagonal) using Cavalli-Sforza and Edwards (1967) among all locations (labelled as in Figure 1).

Sample	PEL	TIC	PRI	ADI	BRE	PIA	TAG	ISO	PRE
**PEL**	***	0.325*	0.529*	0.285*	0.415*	0.182*	0.365*	0.263*	0.648*
**TIC**	0.084*	***	0.338*	0.130*	0.266*	0.174*	0.285*	0.176*	0.481*
**PRI**	0.155*	0.121*	***	0.153*	0.305*	0.336*	0.363*	0.399*	0.502*
**ADI**	0.097*	0.069*	0.059*	***	0.090*	0.100*	0.161*	0.169*	0.223*
**BRE**	0.110*	0.108*	0.118*	0.066*	***	0.115*	0.153*	0.249*	0.314*
**PIA**	0.066*	0.078*	0.125*	0.057*	0.038*	***	0.166*	0.065*	0.337*
**TAG**	0.086*	0.112*	0.163*	0.084*	0.086*	0.054*	***	0.151*	0.221*
**ISO**	0.066*	0.071*	0.162*	0.091*	0.105*	0.053*	0.028*	***	0.495*
**PRE**	0.157*	0.146*	0.166*	0.094*	0.078*	0.084*	0.054*	0.076*	***

*p < 0.001

**Table 4:** Genetic differentiation (*F*_ST_) using Hierarchical AMOVA includes *F*_CT _(among groups) and *F*_SC _(within groups).

Grouping	***F***_**ST**_	***F***_**CT**_	***F***_**SC**_
2 (Western/Central-Italy; Eastern-Italy/Slovenia)	0.244 (p = 0.000)***	0.013 (p = 0.430)	0.231 (p = 0.000)***
3 (Western/Central-Italy; Eastern-Italy; Slovenia)	0.237 (p = 0.000)***	0.003 (p = 0.321)	0.234 (p = 0.000)***
3 (Western-Italy; Central Italy; Eastern Italy/Slovenia)	0.231 (p = 0.000)***	0.078 (p = 0.053)	0.167 (p = 0.000)***
4 (Western-Italy; Central Italy; Eastern Italy; Slovenia)	0.252 (p = 0.000)***	0.098 (p = 0.025)*	0.171 (p = 0.000)***

Groups considered: Western-Italy (Pellice), Central-Italy (Prissiano, Adige, Ticino), Eastern-Italy (Brenta, Piave, Isonzo, Tagliamento), Slovenia (Predelica).

Two different approaches were used to test for possible bottleneck episodes (Table 5), as suggested by the discontinuous allele distribution observed at most loci. However, both softwares (BOTTLENECK and M_P_VAL) suggested demographically stable populations, with no bottleneck signatures observed at any of the samples. Finally, estimation of effective population size using an Approximate Bayesian Calculation (ABC) approach inferred mean *N*_e _values ranging roughly between 2000 and 9000 individuals, with the exception of Predelica, for which a mean *N*_e _value of 858 individuals was inferred (Table 5).

**Table 5:** Results of the bottleneck analyses using BOTTLENECK (Piry *et al*. 1999) and M_P_VAL (Garza and Williamson, 2001), and estimates of effective population size (N_e_) using DIY-ABC (Cornuet *et al*. 2008) including 95% confidence interval.

	BOTTLENECK	M_P_VAL		DIY-ABC	
**Sample**	**p**	**M**	**p**	***N*e**	**95% CI**

**PEL**	1.000	0.76	0.333	3370	196 - 10700
**TIC**	0.813	0.81	0.831	4510	341 - 16100
**PRI**	0.969	0.66	0.137	3110	232 - 10600
**ADI**	0.969	0.83	0.602	9060	604 - 25400
**BRE**	0.130	0.81	0.850	2260	156 - 9360
**PIA**	0.984	0.74	0.178	8240	538 - 28600
**TAG**	0.938	0.76	0.474	3970	308 - 13900
**ISO**	0.813	0.83	0.895	2210	184 - 7600
**PRE**	1.000	0.83	0.922	858	131 - 2790

Samples labelled as in Figure 1.

## Discussion

### Introgression at the mitochondrial and microsatellite level

Mitochondrial data confirmed the existence of genetic introgression from nonindigenous brown trout into marble trout, suggested by previous mitochondrial DNA polymorphism studies [17,20], likely consequence of restocking practices. Presence of non-marble trout haplotypes was highly variable across samples, and included (i) pure marble trout populations (Pellice, Predelica), (ii) low introgression of one or two individuals per population in the North-central locations (Prissiano, Adige, Brenta, Piave and Tagliamento), and (iii) moderate to high introgression (from 11% to 55%) in the remaining populations (Ticino, Adda, Isonzo, Koritnica). By comparison, introgression at nuclear (microsatellite) level was much higher and occurred in all locations except Predelica, reaching extreme levels of introgression in Adda and especially Koritnica, where all individuals were introgressed. A general correspondence was observed between mitochondrial-nuclear introgression, so that locations with no mitochondrial introgression showed <3.5% nuclear introgression, locations with a few introgressed individuals at the mitochondrial introgression showed moderate nuclear introgression (up to 20%), while locations with high introgression at the mitochondrial level (>45%) showed all or practically all individuals introgressed at the nuclear level.

When comparing results with other studies using mitochondrial data, the study of Giuffra et al. [17] with partial sampling overlap with our study (Pellice, Ticino, Brenta) reported a smaller amount of introgression after observing 6 out of 8 pure marble trout populations, which might be due to the lower sampling sizes (N = 8-12). While both studies agree with populations from the western side of the Po river at Pellice being pure marble trout, our study suggested a smaller introgression (7.5%) at Brenta in comparison with the 25% reported in the study of Giuffra et al. [17]. Furthermore, the latter study found no signatures of introgression at Ticino, while our study found 30% exogenous haplotypes. In addition, our sampling overlaps partially with the study of Meraner et al. [20] focusing on the Adige region in South Tyrol (Northern Italy), which reported a higher introgression with all locations showing >20% exogenous haplotypes. By contrast, the two locations examined in our study from the Adige river system only showed 1 out of 30 (Prissiano) and 1 out of 72 (Adige) exogenous haplotypes. The differences across studies might be due to the different tributaries or river sections sampled, and/or to change of stocking practices along time.

Our study also identified for the first time the presence of introgressed marble trout populations in the river Soca drainage off Slovenia both at the mitochondrial and nuclear level. While our data confirmed the Predelica sample to be pure marble trout in accordance with Snoj et al. [15] and Fumagalli et al. [16], introgression was evident at Koritnica (about only 15 km from Predelica) and Isonzo (on the Italian side of the Soca river).

The majority of exogenous mitochondrial haplotypes observed in our marble trout data corresponded to the allochthonous Atlantic haplotype AT-S1. The occurrence of Atlantic haplotypes is unlikely to result from natural migration and might be the consequence of restocking practices. The AT-S1 haplotype group is found at high frequencies in hatchery populations and is thought to represent the predominant haplotype in commercial hatchery strains throughout southern Europe [22,23]. Massive restocking has been conducted in the Po river and its tributaries, to the extent that hybrids with intermediate morphometric traits between brown and marble trout are common [17,18,22]. Similarly, the presence in high frequencies of the nonindigenous Danubian haplotype DA-S1 in the samples of the Ticino region (23-45%) might be consequence of the documented translocations of individuals from Soca river (Slovenia) to Ticino and Adda rivers for restocking purposes [22]. This might also explain (i) the presence of the marble trout MA-S1 haplotype in Ticino, which is typical of the Slovenia region and absent west off Piave, and (ii) the presence in Ticino and Adda of the exclusive marble trout haplotype MA-S4, which is more similar to the Slovenian haplotype (MA-S1) than to the West Italian haplotype (MA-S2). Alternatively, a natural colonisation of Danubian individuals carrying the DA-S1 haplotype due to temporary connections between basins during the Pleistocene cannot be ruled out. It is known that during the Lago Mare period following the Messinian salinity crisis many exchanges and dispersions of brackish and freshwater fish fauna occurred [24]. A similar scenario has been suggested by Meraner et al. [20] in order to explain the presence of DA-S1 haplotypes in the Adige river, with a possible connection via the Drave river drainage allowing migration between Danubian and Adriatic neighbouring tributaries.

### Genetic Differentiation at micro- and macro-geographic scale

Our study reveals strong genetic differentiation among native populations across the geographical range of marble trout using microsatellite data, which suggests restricted gene flow both at the micro-geographic (within rivers) and macro-geographic (among river systems) scale.

Genetic heterogeneity was found not only between geographically distant rivers but also within the same river basin. Samples collected no more than 15 km apart in tributaries of the same river, for instance Adige and Prissiano, resulted significantly differentiated, indicative of limited genetic exchange. However, genetic differentiation in our study was much lower in comparison with the study of Fumagalli et al. [16] on Slovenian marble trout. The authors found extreme *F*_ST _values between samples using microsatellite loci, with an average pairwise *F*_ST _value of 0.79 (about three times higher than the average pairwise *F*_ST _value found in our study of 0.276), indicative of complete absence of genetic exchanges. They suggested the remnant marble trout populations from Slovenia to be completely isolated from each other and from the main river system as immigration from downstream is precluded by impassable barriers. Furthermore, in the populations analyzed by Fumagalli et al. [16], microsatellite allele numbers (1.2-2.0) and heterozygosities (*H*_e _= 0.04- 0.22) were remarkably low, pointing to possible past or present bottleneck episodes. By comparison, most populations in our study showed expected heterozygosities up to 0.388 and allelic richness ranging between 2.3 and 3.4, with the only exception being Predelica (*H*_e _= 0.08; AR = 1.4), a location in the Soca river in Slovenia shared with the study of Fumagalli et al. [16]. Concordantly, an Approximately Bayesian Calculation approach suggested the smallest effective population size for Predelica (*N*_e _= 858). The fact that the rest of locations in our study showed (i) moderate to high genetic diversity, (ii) no signatures of a genetic bottleneck, (iii) and an inferred effective population size over 2000 individuals in all cases suggests that marble trout population in Northern Italy are not extremely isolated and small, unlike the secluded headwater streams of the Soca river.

At the macro-geographic level microsatellite data reveal a highly significant genetic differentiation consistent with a pattern of Isolation-by-Distance. A significant correlation was found between genetic and hydrographic distances that suggests a reduction in gene flow among populations directly related to the increase of waterway distances among sampling locations. According to the Isolation-by-Distance effect, positioning of samples on the horizontal axis of the Multi-Dimentional Scaling plots (Figure 3) clearly reflected the West-to-East geographic distribution of the samples. This pattern was supported by the distribution of mitochondrial haplotypes, with haplotype MA-s2 being fixed in all tributaries from Eastern and Central Italy and gradually decreasing in frequency moving toward Slovenian rivers, where it is replaced by haplotype MA-s1.

It is generally assumed that the intra-specific genetic structure largely reflects the timing and the pattern of habitat colonisation across the entire range distribution of the species. This holds true especially for freshwater species, for which colonisation patterns have been strongly determined by the geological evolution of the hydrographic networks in the past. The Wurmian glacial period (with a maximum peak 18000 years ago) is thought to have played a fundamental role in determining habitat expansion and contraction of most of native freshwater species in the North-Italian and North-western Balkan regions [25]. During the last glacial age, the river Po extended its natural palaeocourse beyond the "meso-Adriatic" sea pit, gathering in the same drainage all the main rivers of the north-east Italian (rivers Tagliamento, Piave, Sile, Brenta and Adige and their hydrographic networks) and Balkan (river Soca) districts. Such a scenario, with a single non-fragmented freshwater basin, suggest an initial common gene pool for all the North-Italian/Slovenian marble trout populations. At the beginning of the postglacial period, the receding of the coastline determined a progressive submerging of the relict outlet of the Po river. This process sequentially isolated the outlet of the rivers presently flowing into the North Adriatic Sea, likely determining a strong reduction of the gene flow among populations confined in different river basins. As a consequence of confinement, individuals tended to find mates from the same or nearby populations rather than distant populations, and as a result, populations that live near each other became genetically more similar than populations that live further apart, generating the pattern of Isolation-by-Distance found in our study.

Besides historical patterns of isolation among drainages, the high rates of introgression may have affected the native patterns of population genetic structure. While a similar East-to-West partition of the microsatellite polymorphism following an Isolation-by-Distance pattern and comparable *F*_ST _values were found when including all individuals in the analysis and when including only native (non-introgressed) individuals, some introgression could remain considering that we excluded introgressed individuals using a 10% criterion. In addition to past biogeographic events, the higher introgression observed in Central Italy could contribute to the deep genetic break between Pellice and Ticino, and between Isonzo and Predelica, and the differences within river basins. In regards to the differences in genetic diversity, introgression might have also contributed to a higher native genetic diversity in Central Italy in comparison with the more extreme locations (Predelica, Pellice), but the fact that a similar native genetic diversity is observed in locations with high (Ticino, Brenta) and low introgression (Adige) suggests that introgression alone cannot account for those differences.

Preserving genetic diversity within a population or species sustains its evolutionary potential to cope with current and future environmental changes. In this sense, native species are specially vulnerable to human activities including introduction of nonindigenous species. Loss of genetic integrity as a consequence of introgression of exogenous gene pools is considered the most serious thread to native gene pools. Thus, detecting hybridization and introgression in wild populations is a primary concern in developing conservation and management strategies. The risk of genetic erosion caused by introgression cannot be ignored as it obscures native gene pools through alteration of genetic composition, reduction of fitness, diminution of population sizes, loss of genetic diversity and extinction of local populations.

## Materials and methods

A total of 429 marble trout *Salmo trutta marmoratus *individuals were caught using electrofishing over the period 1995-1997. Collecting sites included most of the geographic distribution of the species, ranging from North-western Italy to Slovenia (Figure 1). Samples were obtained at the headwaters of eleven tributaries of the Po and Soca (known as Isonzo in Italy) rivers. During field work, fin clips were collected from anaesthetised individuals that were immediately released back to the water. Specimens were photographed and classified as marble trout on the basis of typical morphological features and skin colour patters according to the criteria described by Gandolfi et al. [26]. An additional pure brown trout sample (confirmed at the mitochondrial and microsatellite level) was obtained at the river Chisone in North-West Italy for introgression studies.

DNA was extracted from ethanol-preserved finclips following a resin-based protocol (Chelex 100; Bio-rad) as described in Walsh et al. [27]. A 352 bp segment of the mtDNA control region (D-loop) was amplified using primers LPro2 (5' AACTCCCACCACTAACTCCCAAAGC 3'; [28]) and H2 (5' CGTTGGTCGGTTCTTAC 3'; [29]) in a GeneAmp System 9700 Termocycler (Applied Biosystems). PCR products were used for SSCP (Single Strand Conformation Polymorphism) analysis [30]. Electrophoresis was performed for 40 h in a 12% acrylamide gel at constant room temperature (4°C). DNA fragments were visualized by silver staining. All rare (low frequency) electrophoretic profiles plus a subset of 25 individuals from each common profile were sequenced using a 725 VISTRA automatic sequencer (Molecular Dynamics). Sequences were analyzed using the CHROMAS software (http://www.technelysium.com.au/chromas.html) and aligned using CLUSTALW [31].

All samples were scored for five microsatellite loci: four previously described in brown trout *Salmo trutta*, Str15 [32], Str85, Str543, Str591 [33]; and one previously described in Atlantic salmon *Salmo salar*: Ssa85 [34]. PCR products were obtained in a GeneAmp PCR System 9700 Thermocycler (Applied Biosystems). PCR reactions consisted of 1 μl template DNA, 0.8 μl 10X reaction buffer (Promega), 0.4 μl 1 mM dNTPs, 0.8 μl 25 mM MgCl_2_, 0.3 μl 10 mM forward and reverse primers, 0.04 μl Taq polymerase (Promega, 5 u/μl) and water up to 8 μl. PCR products were run on a 725 VISTRA automatic sequencer (Molecular Dynamics) and analyzed using FRAGMENTOR (Molecular Dynamics).

Analysis of mtDNA data was performed using ARLEQUIN v. 3.5 [35], including computation of haplotype frequencies and exact test of population differentiation. Observed haplotypes were compared with those considered typical of marble trout and brown trout [5] in order to detect possible signatures of introgression. The presence of exogenous DNA material was also tested at microsatellite loci using the software STRUCTURE [36], a model based clustering algorithm that infers the most likely number of groups (species) in the data. The analysis included all marble trout samples from our study plus a sample of brown trout obtained in the river Chisone (North-western Italy, N = 23). The software organizes individuals into a predefined number of clusters (K) with a given likelihood, which may represent putative populations or species. The analysis was performed with 1 < K < 10 to account for population substructuring within species, using the admixture model. The most likely K was determined using the criterion of Evanno et al. [37] and then used to assign each individual. A burn in length of 10^4 ^iterations followed by 10^5 ^additional Markov Chain Monte Carlo (MCMC) iterations were performed as recommended by Pritchard et al. [36].

At microsatellite loci, within-sample genetic diversity statistics were assessed by observed (*H*_o_) and expected (*H*_e_) heterozygosities per locus using GENETIX version 4.05 [38], and allelic richness (*AR*) using FSTAT [39]. Differences in genetic diversity among samples were tested by one-way ANOVA using STATISTICA version 6.0 (StatSoft Inc.). Deviations from Hardy-Weinberg Equilibrium (HWE), linkage disequilibrium, and differences in allele and genotype frequencies among samples were tested using GENEPOP version 3.4 [40]. Presence of null alleles was tested using the program MICRO-CHECKER version 2.2.3 [41].

Prior to the population structure analysis, the statistical power of the markers employed in our study for investigating genetic differentiation was assessed with the program POWSIM [42]. We tested a range of predefined levels of expected divergence (*F*_ST _= 0.001, 0.005, 0.01, 0.05 and 0.1).

Population structure was studied using non-hierarchical and hierarchical *F*-Statistics [43] calculated using ARLEQUIN [35]. Significance tests were assessed with 10,000 permutation tests. In all cases, significance levels were corrected for multiple comparisons using Bonferroni [44]. Pairwise multilocus comparisons between samples were calculated by Cavalli-Sforza and Edwards chord distance [45] and a multivariate ordination was conducted by Multi-Dimensional Scaling (MDS) analysis using STATISTICA version 6.0 (StatSoft). Isolation-by-Distance (IBD) was tested using single and partial Mantel tests implemented in GENETIX, by correlating linearized genetic distance (*F*_ST_/(1- *F*_ST_)) vs. geographic distance (shortest waterway distance between pairs of samples).

The software DIYABC [46] was used to estimate the effective population size for all population samples. The software allows to make inference based on Approximate Bayesian Computation (ABC), in which scenarios can be customized by the user to fit many complex situations involving any number of populations and samples. We estimated effective population size for each population. For all DIYABC runs, prior uniform distributions were set for *Ne *(lower limit 100 and upper limit 100 000), mean *μ *(lower limit 1 × 10^-5 ^and upper limit 1 × 10^-3^) and locus *μ *(lower limit 1 × 10^-5 ^and upper limit 1 × 10^-2^). Microsatellite repeat motif and allele range were specified for each locus. The stepwise mutation model was used and 500,000 replicate runs were performed to generate the reference tables.

Finally, we tested for a genetic bottleneck episode in two different ways. First, we used the software BOTTLENECK [47], based on the principle that after a recent reduction of their effective population size, number of alleles (k) decreases faster than heterozygosity (H_e_) at polymorphic loci. Thus, in a recently bottlenecked population, the observed gene diversity is higher than the expected equilibrium gene diversity (H_eq_) which is computed from the observed number of alleles (k), under the assumption of a constant-size (equilibrium) population [48]. We used the Multiple-step Stepwise (TPM) model [49], which consists of mostly one-step mutations but a small percentage of multi-step changes, and is the recommended model for microsatellite data sets rather than the Infinite Alleles (IAM) or Single-step Stepwise (SMM) models [50]. The proportion of singlestep mutation events was set to 90%, variance = 12%. Observed and expected heterozygosities were compared using a Wilcoxon sign-rank test as suggested by Piry et al. [47]. Second, we calculated M, the mean ratio between number of alleles (k) and range in allele size (r), assuming that during a bottleneck episode k decreases faster than r (M_P_Val; [51]). Hence, the value of M decreases when a population is reduced in size. Average M was calculated across loci and compared with the critical value M_crit _estimated after 10,000 simulations and assuming the population to be at equilibrium. In all simulations, three different values of θ were used (5, 10 and 20). A range of mutation models were examined and conservative values were used for p_s _(frequency of one-step mutations) and Δ_g _(average size of non one-step mutations), p_s _= 0.90 and Δ_g _= 3.5.

## Competing interests

The authors declare that they have no competing interests.

## Authors' contributions

JMP analyzed the data and drafted the manuscript. ANL and MS conducted the molecular analyses, contributed to the data analysis and writing of the manuscript. TP designed and coordinated the study. All authors read and approved the final manuscript.
